# The impact of active smoking on postoperative morbidity and hernia recurrence following abdominal wall reconstruction: long-term follow-up

**DOI:** 10.1007/s10029-026-03625-7

**Published:** 2026-02-27

**Authors:** Nir Messer, Adar Horowitz, Benjamin T. Miller, Lucas R. A. Beffa, Clayton C. Petro, Ajita S. Prabhu, Li-Ching Huang, Eliad Karin, Fahim Kanani, Eran Nizri, Guy Lahat, Amir Szold, Michael J. Rosen

**Affiliations:** 1 Department of Surgery, Gray Faculty of Medicine, Tel Aviv Sourasky Medical Center, Tel -Aviv University, Tel Aviv, Israel; 2 Tel -Aviv University, Tel Aviv, Israel; 3 Department of General Surgery, Cleveland Clinic Center for Abdominal Core Health, Cleveland Clinic Foundation, Cleveland, OH USA; 4 Department of Biostatistics, Vanderbilt University Medical Center, Nashville, TN USA; 5 Assia Medical Group, Assuta Medical Center, Tel Aviv, Israel; 6 Division of GI Surgery, Northwestern University, Chicago, IL USA; 7 Ha-Note’a Street 26, Tel Mond, Israel

**Keywords:** Tobacco smoking, Ventral hernia, Open transversus abdominis release, Surgical site infection, Mesh complications, Hernia recurrence

## Abstract

**Introduction:**

Active smoking is widely regarded as a risk factor for wound morbidity and adverse outcomes in abdominal wall reconstruction (AWR), often serving as a contraindication to elective repair. However, its independent impact on long-term complications remains poorly characterized.

**Methods:**

This study utilized the prospectively maintained ACHQC registry, included patients who underwent open, elective, clean ventral hernia repair (VHR) with transversus abdominis release (TAR) and permanent synthetic mesh at a high-volume AWR center between February 2019 and December 2022. All active smokers during this period were propensity matched in a 1:3 ratio to never-smokers based on demographics, comorbidities, and operative characteristics. Outcomes were assessed at early (30-day) and long-term (≥ 24 months) timepoints. Primary endpoints included wound morbidity, mesh-related complications and hernia recurrence.

**Results:**

A total of 106 active smokers were propensity-matched to 295 never-smokers. Baseline demographics and operative variables were well balanced. At 30 days, there were no significant differences in surgical site infection (SSI) (9.4% vs. 9.2%, *p* = 0.92), surgical site occurrence (SSO) (21.5% vs. 17.6%, *p* = 0.48), or surgical site occurrence requiring procedural intervention (SSOPI) (9.4% vs. 9.2%, *p* = 0.92). At 24 months, rates of SSO (1.9% vs. 0.7%, *p* = 0.81), SSI (0% vs. 0.3%, *p* > 0.99), SSOPI (0% vs. 0.7%, *p* > 0.99), mesh infection (0.9% vs. 0%, *p* > 0.99), and reoperation were low and statistically similar. Hernia recurrence at ≥ 24 months was 4.7% in smokers vs. 7.8% in never-smokers (*p* = 0.15).

**Conclusion:**

In this analysis, active smoking was not associated with increased risk of clinically significant wound morbidity, mesh-related complications, SSOPI, or hernia recurrence. These findings support a patient-centered approach wherein smoking cessation is strongly encouraged but not mandated prior to surgical repair in appropriately selected individuals.

## Introduction

Postoperative wound morbidity, particularly surgical site infection and surgical site occurrences, remains a major focus in abdominal wall reconstruction (AWR) surgeries, given their potential to precipitate mesh-related sequelae, reoperations, and hernia recurrence [1, 2]. Active smoking has been repeatedly linked to increased wound morbidity and thus is commonly considered a contraindication to elective AWR surgeries [3, 4]. This has led many centers to deny operative intervention to active smokers with major ventral hernias [3, 4].

Despite this prevailing paradigm, the actual contribution of active smoking to wound morbidity and late mesh-related complications in modern AWR is not well characterized, as much of the existing evidence derives from large databases, with heterogeneous hernia repair techniques, in multiple settings, and often over-inflates odds ratios with relatively similar effect sizes [5].

In a 2018 study, our group evaluated wound outcomes in patients undergoing elective open ventral hernia repair (VHR) with clean wounds. Our findings demonstrated that active smoking exerted only a minimal impact on clinically meaningful wound morbidity [6]. Consequently, our institution discontinued its mandate for preoperative smoking cessation prior to AWR surgeries, while continuing to educate patients on the broader risks of tobacco use. Subsequently, in 2023, we assessed early postoperative outcomes in patients who underwent open elective AWR with transversus abdominis release (TAR) and synthetic permanent mesh following this policy shift. While active smokers exhibited a modest increase in minor wound morbidity, this did not translate into increased rates of major wound complications or mesh-related adverse events [5].

The present study extends this work by evaluating long-term outcomes among this same cohort, to determine whether this policy shift affects durable clinical endpoints.

## Methods

Following approval from the institutional review board (IRB) at the Cleveland Clinic Foundation, the Abdominal Core Health Quality Collaborative (ACHQC) database was queried for identifying all active-smoker patients from the index study. The ACHQC is a hernia-specific nationwide registry aimed at improving the quality of hernia care through patient-centered data collection, performance feedback to clinicians, and collaborative learning. Surgeons enter patient data prospectively in real-time during care, including patient demographics, hernia characteristics, operative details, patient-reported outcomes (PROs), and postoperative follow-up information [7].

The index study included 106 patients who underwent open, elective VHR with TAR and permanent synthetic mesh between February 2019 and December 2022 and have a minimum of 24 months follow-up. The initial date was chosen to encompass only those patients treated after our policy change of not mandating smoking cessation prior to hernia repair. To ensure a consistent and unambiguous definition of active smoking, patients in the index study were classified into two groups: “active-smokers” (within 30 days of surgery) and “never-smokers” as a control group. These definitions will be maintained in this current study.

Active-smokers were matched using a 1:3 ratio to a group of never-smokers based on propensity scores. We estimated the propensity score with a logistic regression and included the following in the model: age, body mass index (BMI), diabetes mellitus, hernia width, recurrent hernia, and American Society of Anesthesiologists classification (ASA). Nearest neighbor matching with a caliper approach was used. The caliper width was set at 0.2 of the pooled standard deviation of the logit of the propensity score. To assess the balance distribution between active-smokers and never-smokers groups, the standardized mean differences (SMDs) were used. SMD less than 0.1 was considered balanced and less than 0.2 was acceptable. No missing values were observed in the covariates included in the propensity score matching model. Therefore, no missing data imputation methods were applied. Continuous variables were summarized using mean and standard deviation (SD) and comparing between groups using Wilcoxon rank sum test; categorical variables were summarized using frequency and percentage and comparing between groups using Person Chi-squared test. statistical significance was set at 0.05. All statistical analyses were performed using R version 4.4.

Postoperative outcomes were assessed at two intervals: early (at 30 days) and extended (≥ 24 months). Wound morbidity included surgical site infections (SSI), surgical site occurrences (SSO), and surgical site occurrences requiring procedural intervention (SSOPI) [7]. SSIs were further categorized based on the CDC definition into superficial, deep, or organ space infections [8]. SSOs incorporated SSIs and other conditions such as wound cellulitis, non-healing incisional wounds, fascial disruption, skin or soft tissue ischemia, necrosis, serous or purulent wound drainage, stitch abscess, seroma, hematoma, infected or exposed mesh, or the development of an enterocutaneous fistula [7]. SSOPI incorporated any SSO that requires opening of the wound, wound debridement, excision of sutures, percutaneous drainage, or partial or complete mesh removal [7]. Additionally, 30-day all morbidity including ileus, bowel obstruction, pulmonary embolism (PE), stroke, deep venous thromboembolism (DVT), myocardial infarction (MI), cardiac arrest, sepsis, septic shock, pneumonia, urinary tract infection (UTI), acute kidney injury (AKI), renal failure, re-intubation, and other complications were also reported.

Long-term outcomes were evaluated at least 24 months (± 4 weeks) following hernia repair and included readmission, intra-abdominal complications (e.g., fluid collections, hematoma, abscess), mesh infections, various fistulas development, reoperation, mesh excision, and pragmatic hernia recurrence. Pragmatic hernia recurrence was determined based on prior work published by Krpata et al. [9]. In brief, clinical follow-up comprising surgeon evaluation and/or CT scans were employed to determine recurrence. If clinical and imaging evaluation did not occur, recurrence evaluation was carried out utilizing the Hernia Recurrence Inventory (HRI) [10]. In cases where a bulge was reported on HRI, the imaging could overrule that finding. If no other evaluation occurred, a bulge was considered a recurrence.

Long term quality of life was assessed utilizing the hernia-related quality of life score (HerQLes) and National Institutes of Health Patient-Reported Outcome Measurement Information System (PROMIS) Pain Intensity 3 A T-Score. HerQLes is a validated patient-reported outcomes measure consisting of 12 questions focused on the impact of an individual’s abdominal wall on their quality of life [11]. Modified HerQLes scores are calculated on a scale of 0 to 100, wherein higher HerQLes summary scores signify an enhanced quality of life. The minimum clinical important difference (MCID) of the HerQLes score has been determined at 15.6 [12]. The PROMIS Pain Intensity Short Form 3 A is a validated assessment tool encompassing three questions about patients’ pain over the preceding 7 days. It is rated between 30.7 and 71.8, with a value of 30.7 signifying an absence of pain, while higher scores correspond to escalated pain levels [13]. MCID of PROMIS score has not been determined yet for ventral hernias. Change from baseline scores for these measures was calculated at 12-months (± 4 weeks) and at 24-month (± 4 weeks).

## Results

From February 2019 to December 2022, a total of 922 patients underwent open, elective VHRs with TAR at the Cleveland Clinic Center for Abdominal Core Health and completed 30-day and at least 24-month follow-up. Among these, 106 active smokers were matched in a 1:3 fashion with 295 never-smokers. The matched cohorts were comparable in baseline demographics and comorbidities, with the exception of a higher prevalence of COPD among smokers [24 (22.6%) vs. 11 (3.7%), *p*<0.001), and higher rates of anticoagulant use among never-smokers [3 (2.8%) vs. 28 (9.5%), *p*=0.047) (Table 1).

**Table 1:** 1:3 propensity matched demographics and comorbidities characteristics

Demographics and comorbidities	Active Smokers	Never Smokers	Total population	*P*-value
	*N* = 106	*N* = 295	*N* = 401	
Age, y (mean ± SD)	55.6 (± 10.6)	56.1 (± 11.9)	56 (± 11.6)	0.675
Female	49 (46.2%)	162 (54.9%)	211 (52.6%)	0.155
BMI (mean ± SD)	31.1 (± 5.5)	31.5 (± 5.4)	31.4 (± 5.4)	0.522
Hypertension	66 (62.3%)	174 (59.0%)	240 (59.9%)	0.634
COPD	24 (22.6%)	11 (3.7%)	35 (8.7%)	< 0.001
Liver Failure	1 (0.9%)	4 (1.4%)	5 (1.2%)	> 0.99
Diabetes Mellitus	18 (17.0%)	47 (15.9%)	65 (16.2%)	0.922
Inflammatory bowel disease	6 (5.7%)	27 (9.2%)	33 (8.2%)	0.36
Crohn’s disease	6	14	20	0.085
Ulcerative colitis	0	13	13	
Immunosuppressants	7 (6.6%)	29 (9.8%)	36 (9.0%)	0.424
Anti platelet medications	13 (12.3%)	18 (6.1%)	31 (7.7%)	0.068
Anticoagulant medications	3 (2.8%)	28 (9.5%)	31 (7.7%)	0.047
ASA class				
1	0 (0%)	0 (0%)	0 (0%)	0.658
2	19 (17.9%)	54 (18.3%)	73 (18.2%)	
3	86 (81.1%)	234 (79.3%)	320 (79.8%)	
4	1 (0.9%)	7 (2.4%)	8 (2.0%)	

*ASA* American Society of Anesthesiologists; *VHWG* Ventral Hernia Working Group

Hernia characteristics and operative variables are summarized in Table 2. No statistically significant differences were identified. 398 (99.3%) cases involved incisional hernias (*p* > 0.99), and the proportion of patients with a history of recurrent hernia was similar between groups [47 (44.3%) vs. 139 (47.1%), *p*=0.705). Mean hernia width was 14.5 cm (± 5.5) in the active smokers group and 14.7 cm (± 5.8) in never-smokers (*p*=0.895), and mean mesh width was 34.5 ± 9.0 cm and 33.9 ± 8.1 cm, respectively (*p*=0.262). With a mean hernia width of 14.5 cm and a mean mesh width of 34 cm, an average lateral overlap of approximately 10 cm was achieved on each side. Primary fascial closure was achieved in 103 patients (97.2%) of the active smokers group and 281 (95.3%) of the never-smokers group (*p*=0.576). Additional hernia and operative details are provided in Table 2.

**Table 2:** 1:3 propensity matched hernia and operative characteristics

Hernia and operative characteristics	Active Smokers	Never Smokers	Total population	*P*-value
	*N* = 106	*N* = 295	*N* = 401	
Hernia type				
Incisional	105 (99.1%)	293 (99.3%)	398 (99.3%)	> 0.99
Parastomal hernia	0 (0%)	3 (1%)	3 (0.7%)	0.7
Recurrent hernia	47 (44.3%)	139 (47.1%)	186 (46.4%)	0.705
Number of prior hernia repairs (mean ± SD)	2.2 (± 1.3)	2 (± 1.2)	2.1 (± 1.3)	0.489
Stoma present	0 (0%)	4 (1.4%)	4 (1%)	0.525
History of component separation prior to current AWR	7 (6.6%)	25 (8.5%)	32 (8%)	0.689
History of open abdomen prior to current AWR	11 (10.4%)	19 (6.4%)	30 (7.5%)	0.269
History of prosthetic mesh infection prior to current AWR	14 (13.2%)	15 (5.1%)	29 (7.2%)	0.237
Previously infected mesh removed prior to current AWR	13 (12.3%)	14 (4.7%)	27 (6.7%)	> 0.99
Hernia length, cm (mean ± SD)	21.6 (5.7)	21.6 (5.7)	21.6 (5.7)	0.764
Hernia width, cm (mean ± SD)	14.5 (5.5)	14.7 (5.8)	14.6 (5.7)	0.895
Mesh length, cm (mean ± SD)	34.3 (8.5)	33.9 (8)	34.1 (8.1)	0.309
Mesh width, cm (mean ± SD)	34.5 (9)	33.9 (8.1)	34.1 (8.3)	0.262
Fascial closure	103 (97.2%)	281 (95.3%)	384 (95.8%)	0.576
Drain Placement				
Retromuscular	102 (96.2%)	293 (99.3%)	395 (98.5%)	0.074
Intraperitoneal	1 (0.9%)	1 (0.3%)	2 (0.5%)	> 0.99
Operative time>2 h	88 (83%)	245 (83.1%)	333 (83%)	> 0.99

30-day postoperative wound and overall morbidity outcomes are summarized in Table 3. Total SSO and SSI rates were similar between groups. The total SSO rate was 20 (21.5%) in active smokers group and 49 (17.6%) in never-smokers (*p* = 0.48). The total SSI rate was 10 (9.4%) in active smokers and 27 (9.2%) in never-smokers (*p* = 0.92). Further stratification revealed no significant differences in rates of superficial, deep, or organ space infections (Table 3). The incidence of noninfected seroma was significantly higher in the active smokers group, occurring in 8 patients (7.5%) versus 3 (1.0%) in the never-smokers (*p* < 0.001). Other wound-related complications were infrequent and not statistically significant. The rate of SSOPI was comparable between groups with 10 cases (9.4%) in active smokers vs. 27 cases (9.2%) in never-smokers (*p* = 0.92). Reoperation was required in 11 patients (2.7%) overall, including 2 (1.9%) active smokers patients and 9 (3.1%) never-smokers (*p* = 0.76). No cases of mesh removal or deaths were reported. One notable postoperative complication was pneumonia, which occurred more frequently in smokers [5, (4.7%)] than in never-smokers [3, (1.0%)], though this difference did not reach statistical significance (*p* = 0.053).

**Table 3:** 1:3 propensity matched 30-day wound morbidity and clinical outcomes

Wound morbidity and clinical outcomes	Active Smokers	Never Smokers	Total population	*P*-value
	*N* = 106	*N* = 295	*N* = 401	
SSO	20 (21.5%)	49 (17.6%)	69 (18.5%)	0.488
Superficial SSI	10 (9.4%)	15 (5.1%)	25 (6.2%)	0.12
Deep SSI	0 (0%)	10 (3.4%)	10 (2.5%)	0.139
Organ space SSI	0 (0%)	2 (0.7%)	2 (0.5%)	> 0.99
Total SSI	10 (9.4%)	27 (9.2%)	37 (9.2%)	0.92
Wound cellulitis	2 (1.9%)	7 (2.4%)	9 (2.2%)	> 0.99
Wound purulent drainage	0 (0%)	0 (0%)	0 (0%)	N/A
Stitch abscess	0 (0%)	0 (0%)	0 (0%)	N/A
Infected mesh	0 (0%)	0 (0%)	0 (0%)	N/A
Noninfected Seroma	8 (7.5%)	3 (1%)	11 (2.7%)	< 0.001
Infected seroma	0 (0%)	1 (0.3%)	1 (0.2%)	> 0.99
Wound serous drainage	1 (0.9%)	5 (1.7%)	6 (1.5%)	> 0.99
Hematoma	1 (0.9%)	2 (0.7%)	3 (0.7%)	> 0.99
Infected hematoma	0 (0%)	0 (0%)	0 (0%)	
Non-healing incisional wound	1 (0.9%)	3 (1%)	4 (1%)	> 0.99
Fascial disruption	0 (0%)	1 (0.3%)	1 (0.2%)	> 0.99
Skin or soft tissue necrosis	0 (0%)	4 (1.4%)	4 (1%)	0.562
Exposed mesh	1 (0.9%)	2 (0.7%)	3 (0.7%)	> 0.99
SSOPI	10 (9.4%)	27 (9.2%)	37 (9.2%)	0.92
Wound opening	5 (4.7%)	7 (2.4%)	12 (3%)	0.309
Wound debridement	0 (0%)	4 (1.4%)	4 (1%)	0.562
Percutaneous drainage	0 (0%)	1 (0.3%)	1 (0.2%)	> 0.99
Partial mesh removal	0 (0%)	0 (0%)	0 (0%)	N/A
Complete mesh removal	0 (0%)	0 (0%)	0 (0%)	N/A
Reoperation	2 (1.9%)	9 (3.1%)	11 (2.7%)	0.763
Re-intubation for respiratory failure	2 (1.9%)	4 (1.4%)	6 (1.5%)	> 0.99
Pneumonia	5 (4.7%)	3 (1%)	8 (2%)	0.053
UTI	3 (2.8%)	2 (0.7%)	5 (1.2%)	0.229
DVT	1 (0.9%)	2 (0.7%)	3 (0.7%)	> 0.99
Pulmonary embolism	1 (0.9%)	5 (1.7%)	6 (1.5%)	0.936
Stroke	1 (0.9%)	0 (0%)	1 (0.2%)	0.593
Myocardial infarction	0 (0%)	0 (0%)	0 (0%)	N/A
Cardiac arrest	0 (0%)	0 (0%)	0 (0%)	N/A
Deceased at 30 days	0 (0%)	0 (0%)	0 (0%)	N/A
Length of stay (mean ± SD)	6.4 (± 6.7)	6.2 (± 3.6)	6.2(± 4.6)	0.087
Readmission	6 (5.7%)	24 (8.1%)	30 (7.5%)	0.517

At 24-month follow-up, the overall rate of SSO remained low and did not differ significantly between groups. SSOs were observed in 2 (1.9%) of active smokers and 2 (0.7%) of never-smokers (*p* = 0.81). Seroma rates were identical across cohorts, with 1 case (0.9%) in active smokers and 1 case (0.3%) in never-smokers (*p* = 0.57). One patient (0.3%) in the never-smokers group developed SSI, which was successfully managed with local wound opening (*p* > 0.99). Additionally, one (0.9%) active smoker patient experienced a mesh infection that was treated successfully with antibiotic therapy (*p* > 0.99). The rate of SSOPI was negligible and statistically equivalent in both groups (*p* > 0.99). No incidence of mesh removal or reoperation was documented. Detailed outcomes are provided in Table 4.

**Table 4:** 1:3 propensity matched 24-month wound morbidity

Wound morbidity and clinical outcomes	Active Smokers	Never Smokers	Total population	*P*-value
	*N* = 106	*N* = 295	*N* = 401	
SSO	2 (1.9%)	2 (0.7%)	4 (1%)	0.81
Superficial SSI	0 (0%)	1 (0.3%)	1 (0.2%)	> 0.99
Deep SSI	0 (0%)	0 (0%)	0 (0%)	NA
Organ space SSI	0 (0%)	0 (0%)	0 (0%)	NA
Total SSI	0 (0%)	1 (0.3%)	1 (0.2%)	> 0.99
Wound cellulitis	0 (0%)	0 (0%)	0 (0%)	NA
Wound purulent drainage	0 (0%)	0 (0%)	0 (0%)	NA
Stitch abscess	0 (0%)	0 (0%)	0 (0%)	NA
Infected mesh	1 (0.9%)	0 (0%)	1 (0.2%)	> 0.99
Seroma	1 (0.9%)	1 (0.3%)	2 (0.5%)	0.577
Infected seroma	0 (0%)	0 (0%)	0 (0%)	NA
Wound serous drainage	0 (0%)	0 (0%)	0 (0%)	NA
Hematoma	0 (0%)	0 (0%)	0 (0%)	NA
Infected hematoma	0 (0%)	0 (0%)	0 (0%)	NA
Non-healing incisional wound	0 (0%)	0 (0%)	0 (0%)	NA
Fascial disruption	0 (0%)	0 (0%)	0 (0%)	NA
Skin or soft tissue necrosis	0 (0%)	0 (0%)	0 (0%)	NA
Exposed mesh	0 (0%)	0 (0%)	0 (0%)	NA
SSOPI	0 (0%)	1 (0.7%)	1 (0.6%)	> 0.99
Wound opening	0 (0%)	1 (0.7%)	0 (0%)	> 0.99
Wound debridement	0 (0%)	0 (0%)	0 (0%)	NA
Percutaneous drainage	0 (0%)	0 (0%)	0 (0%)	NA
Partial mesh removal	0 (0%)	0 (0%)	0 (0%)	NA
Complete mesh removal	0 (0%)	0 (0%)	0 (0%)	NA
Reoperation	0 (0%)	0 (0%)	0 (0%)	NA

Long-term hernia recurrence data, with a minimum follow-up of 24 months, are summarized in Table 5. The mean follow-up duration was 29 months. The total cumulative pragmatic recurrence rate at a minimum of 24 months was twelve patients (11.3%) in active smokers and 48 (16.3%) in never-smokers (*p* = 0.22). Within the first 12 months postoperatively, pragmatic recurrence was identified in 7 patients (6.6%) of active smokers and 25 (8.5%) of never-smokers (*p* = 0.81). Between 12 and a minimum of 24 months, an additional 5 patients (4.7%) in the smoking cohort and 23 patients (7.8%) in the never-smoking cohort experienced pragmatic hernia recurrence (*p* = 0.15). No statistically significant differences were observed between groups at any time point.

**Table 5:** 1:3 propensity matched 24-month recurrence

Long term hernia recurrence	Active Smokers	Never Smokers	Total population	*P*-value
	*N* = 106	*N* = 295	*N* = 401	
12-month follow-up				
Patient reported recurrence	12 (11.3%)	53 (18%)	65 (16.2%)	0.38
Clinical recurrence	1 (0.9%)	3 (1%)	4 (1%)	> 0.99
Pragmatic recurrence	7 (6.6%)	25 (8.5%)	32 (8%)	0.81
24-month follow-up				
Patient reported recurrence	4 (3.8%)	21 (7.1%)	25 (6.2%)	0.29
Clinical recurrence	3 (2.8%)	2 (0.7%)	5 (1.2%)	> 0.99
Pragmatic recurrence	5 (4.7%)	23 (7.8%)	28 (7%)	0.15
24-Month Cumulative pragmatic recurrence	12 (11.3%)	48 (16.3%)	60 (14.9%)	*p* = 0.22

Quality of life was assessed using the HerQLes and PROMIS Pain 3 A T-scores at baseline, 12 months, and 24 months (Table 6). No statistically significant differences in HerQLes scores were observed between active smokers and never-smokers at any time point. PROMIS Pain 3 A T-scores indicated a trend toward higher baseline pain in the smoking cohort, with mean scores of 49.0 ± 10.5 in active smokers versus 45.6 ± 10.1 in never-smokers (*p* = 0.007). At 24 months, this trend persisted, with active smokers reporting higher pain scores (44.2 ± 9.1 vs. 38.8 ± 9.4), although the difference did not reach statistical significance (*p* = 0.054).

**Table 6:** Life quality assessment

Life Quality assessment	Active Smokers	Never Smokers	Total population	*P*-value
	*N* = 106	*N* = 295	*N* = 401	
HerQLes score (mean ± SD)				
Baseline	33 (± 25.8)	37.9 (± 25.8)	36.7 (± 25.9)	0.12
12-month	68.6 (± 30.4)	71.5 (± 26.1)	71.1 (± 26.7)	0.87
24-month	64.3 (± 32.5)	77.8 (± 23.6)	76.8 (± 24.4)	0.29
PROMIS Pain 3 A T-score (mean ± SD)				
Baseline	49 (± 10.5)	45.6 (± 10.1)	46.5 (± 10.3)	0.007
12-month	40.2 (± 10.6)	38.8 (± 9.7)	39 (± 9.9)	0.49
24-month	44.2 (± 9.1)	38.8 (± 9.4)	39.2 (± 9.4)	0.054

## Discussion

This study evaluated the short and long-term associations between active smoking and postoperative outcomes, including wound morbidity, mesh-related complications, need for procedural intervention, and hernia recurrence in patients undergoing elective, clean, open ventral hernia repair with transversus abdominis release and permanent synthetic mesh. Consistent with our prior work, which demonstrated no clinically meaningful differences in short-term wound morbidity between active smokers and never-smokers, the current analysis showed that, aside from a higher incidence of noninfected seromas among smokers (7.5% vs. 1.0%, *p* < 0.001), no statistically significant differences were observed in long-term wound morbidity, mesh-related morbidity, SSOPI, or reoperation rates. Given the potential association between wound morbidity and hernia recurrence, it is noteworthy that recurrence rates at a minimum follow-up of two years were also statistically comparable between groups. These findings suggest that active smoking may not independently predict adverse long-term outcomes in this operative context.

Our results challenge a long-standing narrative in the literature that identifies active smoking as a contraindication to elective hernia repair. Many of the studies supporting this position are based on large administrative databases, which often include inconsistent definitions of active smoking and heterogeneous surgical populations, ranging from elective to urgent procedures, clean to contaminated fields, and open to minimally invasive approaches, involving varied mesh materials and planes [2, 3, 14, 15]. However, more recent investigations with narrower inclusion criteria have produced conflicting results, highlighting the complexity of this association.

The relationship between smoking and postoperative wound morbidity and mesh-related complications has been investigated in several large database studies. One of the most frequently cited is a retrospective analysis of ACS-NSQIP data, including 169,458 patients stratified by smoking status (defined as tobacco use within one year before surgery) [14]. After propensity score matching, the study reported a 30-day wound morbidity rate of 4.6% in smokers compared to 3.1% in non-smokers (*p* < 0.001). While this study included multiple operative variables and hernia characteristics, its major critique is the vast population size, which could result in minor differences becoming statistically significant. These differences may lack clinical relevance and may only be noticeable in the context of a large-scale patient cohort undergoing surgical intervention and are particularly exaggerated when reporting odds ratios. In contrast, a more recent population-based registry analysis conducted between 2021 and 2023 included 12,233 patients undergoing elective ventral hernia repair. This study reported no significant differences in 30-day complications (2.45% vs. 2.01%, *p* = 0.212), readmission (2.51% vs. 2.46%, *p* = 0.882), or reoperation (1.37% vs. 1.45%, *p* = 0.786) between active smokers and never-smokers [16].

The evaluation of SSOPI provides valuable granularity in distinguishing minor wound morbidity from outcomes requiring procedural intervention. While few studies explicitly report SSOPI rates, our single center findings are closely aligned with those of Petro et al., who analyzed 418 active smokers and 418 never-smokers undergoing elective open VHR with clean wounds within the ACHQC database [6]. While SSO was higher in smokers (12.0% vs. 7.4%, *p* = 0.03), largely due to increased seroma rates (5.5% vs. 1.2%), no significant differences were observed in SSI (4.1% vs. 4.1%, *p* = 0.98), SSOPI (6.2% vs. 5.0%, *p* = 0.43), or reoperation (1.9% vs. 1.2%, *p* = 0.39). These findings reinforce the conclusion that although smoking may contribute to minor, self-limiting wound events, it does not appear to increase the incidence of major complications requiring intervention.

Regarding hernia recurrence, few studies have evaluated the long-term impact of tobacco smoking with rigorous follow-up. One retrospective analysis using a national registry included 1,307 patients with a mean follow-up of one year and was originally designed to compare urgent versus elective hernia repairs [17]. A subgroup analysis identified smoking as a risk factor for recurrence (OR 2.94; 95% CI: 1.33–9.15, *p* = 0.011). However, the study included both primary and mesh repairs, utilized multiple mesh planes, and reported that only 8% of patients underwent sublay repair, despite 31% requiring component separation. Given the established superiority of the sublay approach in large hernias, the observed recurrence rates may reflect selection bias or technical heterogeneity. By contrast, our study employed a highly standardized operative approach, with all patients undergoing open TAR with permanent polypropylene mesh, and follow-up exceeded 24 months for all included patients, strengthening the internal validity of our recurrence analysis.

Another lens through which to interpret the association between smoking and wound outcomes is the potential benefit of preoperative smoking cessation. While several studies have addressed this, most focus on systemic complications rather than wound-specific morbidity. In a multicenter randomized trial by Lindström et al., 102 patients undergoing various elective surgeries, including hernia repair (42%), cholecystectomy (22%), Hip prosthesis (24%) and Knee prosthesis (33%), were randomized to preoperative smoking cessation or continued smoking. No differences in wound morbidity were found at 30-day follow-up [18]. Similarly, Sørensen et al. randomized 60 daily smokers undergoing colorectal resection to smoking cessation or continued smoking three weeks prior to surgery and found no significant differences in wound or soft tissue complications [19]. In the only known study specifically addressing smoking cessation in the context of AWR, 70 patients undergoing planned AWR with component separation were referred to a prehabilitation program targeting weight loss, glycemic control, and smoking cessation [20]. The median rehabilitation time was 7 months, and the study specifically evaluated 30 and 90-day complications, including: seroma, hematoma, wound infection (superficial, deep and organ space), mesh infection, and flap necrosis, as well as clinical outcomes such as cardiovascular and respiratory morbidity, ileus. Of the 21 patients referred to smoking cessation, only 38% achieved complete abstinence, while the remainder significantly reduced consumption (with a median of 30 cigarettes/day to 7 cigarettes/day). The study findings did not exhibit elevated postoperative wound and soft tissue complication rates compared to the control or published benchmarks. In our study, active smokers reported higher PROMIS Pain 3 A scores, suggesting a greater symptom burden at baseline. While prehabilitation programs may optimize surgical readiness and benefit selected patients, they may also inadvertently delay timely hernia repair in those with severe or disabling symptoms without a demonstrable improvement in surgical outcomes.

Taken together, our findings, alongside recent literature, suggest that a policy of uniformly denying elective hernia repair to active smokers may warrant re-evaluation. While we acknowledge the well-established long-term systemic harm of smoking and continue to counsel patients accordingly, our data do not support a clear association between active smoking and increased risk of major postoperative wound complications or hernia recurrence in the setting of clean, elective abdominal wall reconstruction. Although some may argue that systemic complications, particularly pulmonary events, could justify denying surgery in this population, this rationale should be carefully balanced against the clinical and functional burden of untreated symptomatic hernias. Beyond the risk of incarceration, ventral hernias are known to significantly impair physical function, mobility, and overall quality of life. Left untreated, patients may enter a cycle of worsening disability and diminished surgical candidacy [21–24]. We support a patient-centered approach in which smoking cessation is strongly encouraged and, when feasible, integrated into a preoperative optimization pathway. However, for patients who failed to quit, proceeding with hernia repair may still offer meaningful functional improvement and may help interrupt a cycle of physical debilitation and reduced quality of life, potentially creating an inflection point toward broader health recovery.

This study has several important limitations. First, although data were collected prospectively through the ACHQC, the analysis itself is retrospective in nature and subject to the inherent limitations of observational study design. Second, while our sample size of 106 active smokers and 295 never-smokers is smaller than that of some large database studies, it reflects a focused cohort of patients undergoing elective, clean, elective open TAR for large incisional hernias, an operative context that is both high-risk and clinically relevant. As such, we believe this study provides meaningful insights into this specific patient population, even if not broadly generalizable to all hernia repairs. To establish two clearly defined cohorts, we intentionally excluded former smokers, which may limit external validity, but was necessary to reduce misclassification and enhance the clarity of comparisons. Additionally, our ability to explore a dose–response relationship was constrained by the absence of detailed information regarding cumulative tobacco exposure (e.g., pack-years or intensity of use). Another limitation involves variability in clinical follow-up. Our institutional protocol includes standardized follow-up at 30 days, 12 months, and 5 years, with radiographic assessment (typically CT scan) at 12 and 60 months. However, not all patients adhered to this schedule, and only 86% of the cohort received radiographic imaging. To address this, we performed a comprehensive chart review and directly contacted patients lacking imaging data to obtain follow-up via the HRI, assess for complications, and document reoperations, readmissions, and current health status. Despite these efforts, some degree of outcome misclassification cannot be fully excluded. Additionally, we observed a higher rate of anticoagulant use in never-smokers, which may reflect a different baseline of cardiovascular comorbidities in the control group. While anticoagulant use is a known risk factor for wound events, the SSO and SSI rates remained comparable between groups, suggesting that the impact of active smoking did not exceed the baseline risks present in our matched never-smoker cohort.” It is also important to note that these findings are specific to open TAR and may not be generalizable to abdominal wall reconstructions involving onlay repair or skin flaps, where smoking-related microvascular compromise may have a more pronounced impact. Finally, this study represents a single-center analysis conducted at a high-volume abdominal wall reconstruction program, and therefore, the findings may not be generalized to other institutions or surgical practices.

## Conclusion

**Fig. 1:**
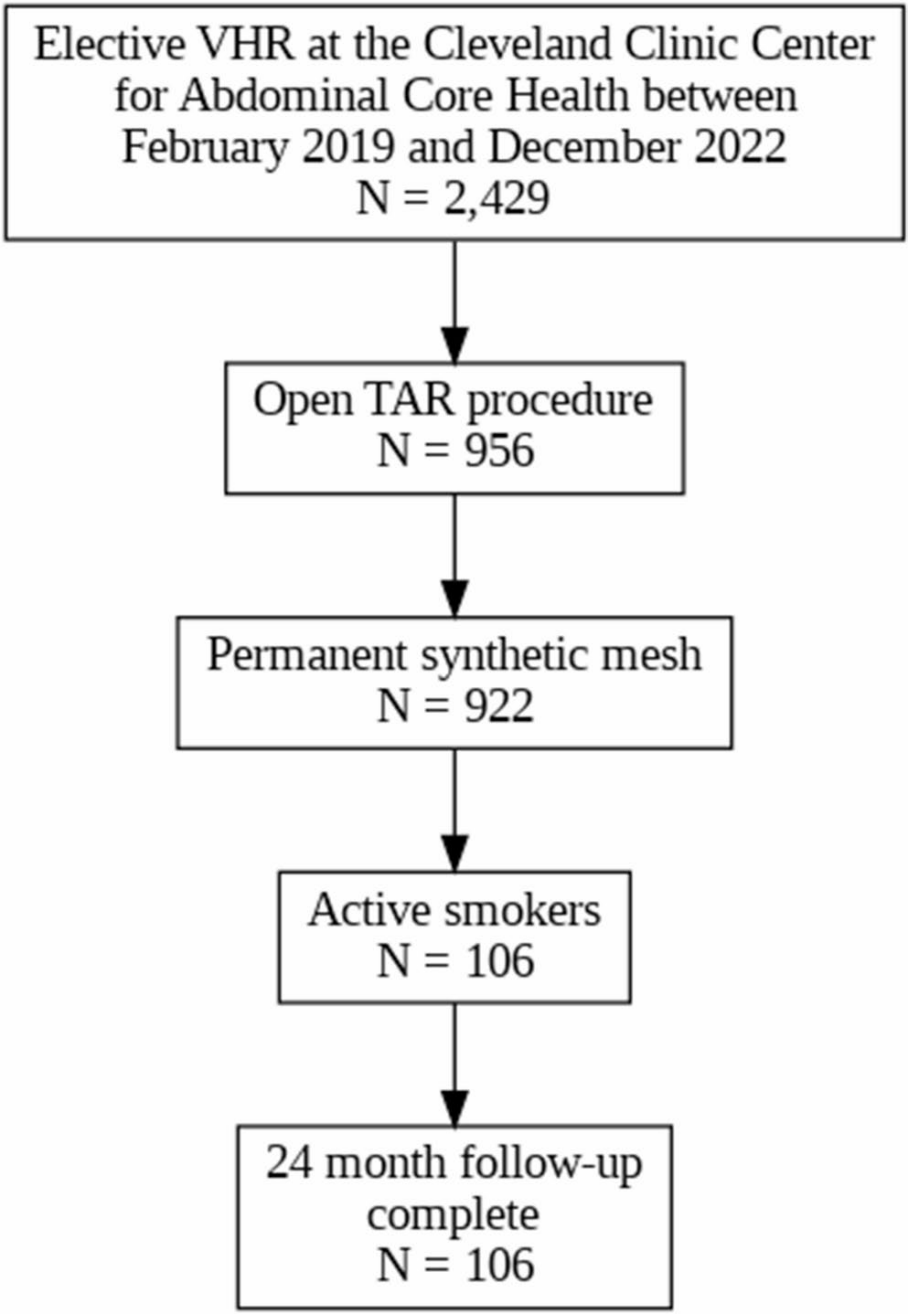
Active smokers who underwent open, elective AWR with TAR and permanent synthetic mesh between February 2019 and December 2022 at the Cleveland Clinic Center for Abdominal Core Health

In this analysis, active smoking was not associated with increased rates of clinically significant wound morbidity, mesh-related complications, SSOPI, or hernia recurrence following elective, open ventral hernia repair with transversus abdominis release and permanent synthetic mesh. While these findings challenge the prevailing assumption that active smoking universally portends worse surgical outcomes, smoking cessation should remain a key component of preoperative counseling. When feasible, cessation should be incorporated into preoperative optimization protocols; however, in patients unable to achieve abstinence, surgical repair should not be categorically withheld (Fig. 1).
